# Young people who inject drugs in Mozambique: should we emphasize them in the National Harm Reduction Plan?

**DOI:** 10.1186/s12954-020-00363-6

**Published:** 2020-03-26

**Authors:** Cynthia Semá Baltazar, Makini Boothe, Timothy Kellogg

**Affiliations:** 1 Instituto Nacional de Saúde, Ministério da Saúde, Maputo, Mozambique; 2 Faculty of Medicine and Health Sciences, Ghent University, Ghent, Belgium; 3 University of California, San Francisco, CA USA

**Keywords:** Harm reduction, Youth, People who inject drugs, Mozambique

## Abstract

Mozambique has one of the highest burdens of HIV globally, and people who inject drugs (PWID) have one of the highest HIV infection rates in Africa. After the implementation of the first Biological Behavioral Surveillance (BBS) Survey among PWID in Mozambique, the Ministry of Health started the development of a National Harm Reduction Plan. Although the findings from the BBS survey highlighted the specific needs of young PWID, the proposed Harm Reduction Plan does not explicitly focus on reducing high-risk behaviors of young PWID. We outline the importance of the inclusion of age-specific interventions focused on the needs of young PWID in Mozambique, and how a comprehensive Harm Reduction Plan can reduce the HIV epidemic in this population. There is a unique opportunity to advocate for the Harm Reduction Plan to include “youth-friendly” cost-effective and evidence-based interventions that are targeted to this important sub-group within an already vulnerable population.

## Background

The period of adolescence and youth aged 10–24 years is typically characterized by experimentation, new experiences, and social vulnerability. Evidence suggests that people who inject drugs (PWID) often begin injecting at a young age [1–3]. Young people face tremendous challenges to access HIV and sexual and reproductive health services, including inequalities, discrimination, exclusion, and violence. Yet, the global response to HIV among PWID largely neglects this critical key sub-population who benefit less than other groups from established service provision models [4]. Drug use behaviors usually emerge as a result of interaction between environmental and social determinants and development process.

Many young PWID do not like accessing health services alongside adult PWID because they feel that they have little in common with the adult PWID who generally access services [1, 2]. Additionally, HIV service providers are often inadequately trained to support youth with sex education, reproductive services, drug use counseling social services, violence prevention, and other youth-specific health concerns [5, 6]. On the other hand, the staff of programs for young people may lack the sensitivity, knowledge, and training to work specifically with youth from key populations [2, 6, 7]. The criminalization of drug use, combined with the high levels of stigma and discrimination related to the illicit nature of this practice, further increases their susceptibility to blood-borne and sexually transmitted infections (STI) and HIV and viral hepatitis (B and C) [1, 3, 8]. The lack of integrated services for HIV, TB, viral hepatitis, STIs, and related to harm reduction hampers the health care seeking in youth population [6]. Neglecting to identify the specific and comprehensive health needs of this key sub-population, and their particular barriers to health care, has the potential to compromise the success of HIV prevention and control programs targeted to those specific populations [4, 9].

Around one-quarter of the world’s population are youth, and nearly 160,000 of new HIV infections globally in 2018 occurred in under 15 age [6, 10]. The overall burden of HIV in the Sub-Saharan African region attributed to injection drug use is limited, and specific data about young PWID remain largely invisible in routine HIV surveillance and surveys. This has a major consequence on the effectiveness of harm reduction policies and programs since they are largely designed for adult PWID [11].

The results from the first Biological Behavioral Surveillance (BBS) survey conducted in 2013–2014 in two urban areas in Mozambique revealed a high prevalence of HIV estimated to be 50.1% (95% CI, 40.1–59.0) in Maputo and 19.9% (95% CI, 10.9–29.2) in Nampula/Nacala [12]. Given this high burden of disease, PWID were recognized by the Mozambique National Strategic HIV/AIDS Response Plan (2015–2019) as a high-risk group for HIV due to their specific risk behaviors, such as sharing contaminated syringes and unprotected sex [13].

After the implementation of the first PWID BBS in Mozambique, the Ministry of Health (MoH) started the development of a National Harm Reduction Plan, under the umbrella of the National Strategy for Mental Health (2016–2026). The main objective of the plan is to address public health problems caused by drug use, reduce risks, and prevent disease. However, thus far, the discussions around the plan’s development have not considered the unique needs of adolescents and young people nor expanded on how the plan could be adapted to ensure that public health services reach this age group.

### International concerns of youth drug use in the HIV epidemic

On a global scale, attention has also focused on improving the quality of services for PWID. There have been various international declarations and commitments with specific goals and targets for young people. In 2016, UN members states adopted the fast track targets aimed to end the global HIV epidemic by 2030. The Political Declaration on HIV and AIDS recognized the critical role of young people in the global HIV epidemic, the inadequate integration of health services, and a set of specific time-frame targets with more young gender-inclusive approach [14]. Although some countries have achieved a general decline in HIV prevalence over the years, such reductions could not be systematically linked to specific programmatic interventions, due to the absence of operational targets and inadequate surveillance systems [15].

The World Health Organization (WHO), United Nations Office of Drugs and Crime (UNODC), and the Joint United Nations Programme on HIV/AIDS (UNAIDS) developed a comprehensive package of Harm Reduction Services for preventing the spread of HIV and reducing other harms associated with drug use. The technical guidance outlines a comprehensive package of interventions for the prevention, treatment, and care of HIV infection among PWID; provides a set of indicators to monitor and evaluate the implementation and impact of these interventions; advises on setting targets for scale-up to maximize the impact of HIV prevention and care among PWID; and provides examples of data sources and useful tools to assist with program development, implementation, monitoring, and evaluation [16].

However, even these various global guiding documents lack specific recommendations for youth, which is a missed opportunity given that these risk behaviors are often developed during adolescence and youth and that the consequences of the behaviors contribute to the burden of disease among this age-group [1, 3, 17].

### Young PWID in Mozambique

Young people, who make up 32.5% of the Mozambican population [18], are a vulnerable sub-population of the country’s HIV epidemic. Data from the population-based 2015 National AIDS Indicator Survey (IMASIDA) results show relatively low levels of knowledge about HIV in young people with 7 in every ten not having a comprehensive knowledge of HIV. Among young men, comprehensive HIV knowledge decreased by 22% since the last household survey conducted in 2011. The HIV prevalence among young people aged 15–24 was 6.9%, where it was higher in young women (9.8% vs. 3.2%). Also, 3% of young women and 18% of young men report having had two or more partners in the last 12 months, and less than 50% of youth reported condom use during their last sexual intercourse. Among participants 15–24 years of age who had sex in the last 12 months, only 38% of women and 18% of men were tested in the last 12 months and received the result of the last test [19]. These vulnerabilities are heightened among young PWID.

Data from the first BBS in Mozambique show that HIV prevalence among PWID aged 18–24 in Maputo was 18.8%. Nearly half of PWID (46.1% for Maputo and 48.2% for Nampula/Nacala) had their first contact with drugs when they were 18–24 years of age. Young PWID also reported high-risk sexual and drug use behaviors. Among sexually active young PWID who injected drugs in the last 12 months, only 44.4% in Maputo and 32.5% of PWID in Nampula/Nacala used a condom during their last intercourse. Less than 70% (66.5% for Maputo and 67.3% in Nampula/Nacala) reported using brand new injection equipment during their last drug injection. Among younger PWID unaware of their status, HIV testing was low, with only 22.4% and 20.2% in Maputo and Nampula/Nacala, respectively, tested for HIV in the 12 months before the survey [20].

### Programmatic and political gaps in services for PWID

The Sub-Saharan Africa region is far behind in implementing and scaling-up harm reduction interventions recommended by the United Nations; similarly, efforts are far below estimates required to reverse the HIV epidemic among PWID in the region [17, 21]. According to the most recent Global State of Harm Reduction publication, only 11 countries have policies that specifically advocate for harm reduction activities. In the specific case of Mozambique, there is only one operational needle and syringe program [22]. The political and legal environment in Sub-Saharan Africa continues to follow a predominantly punitive approach when considering harm reduction, and only a few countries in the region include an explicit supportive reference to harm reduction in their national policies and programs on HIV or drugs [6].

Substance abuse services for PWID in Mozambique are integrated into programs managed by the National Mental Health Program. The 2017 National Mental Health Program Report indicates that 10,120 patients attended the health services due the drug use, of which more than 50% were under 30 years of age [23]; however, current data systems do not specifically outline the age or sex disaggregation of these young patients. During qualitative interviews conducted as part of the BSS, PWID Mozambique listed the co-location of substance abuse services within mental health services as a barrier to treatment because of the stigma associated with seeking psychiatric support [20]. As such, it is likely that less youth access services than are actually in need.

In 2018, the MoH developed the *Integrated Guidelines for Prevention, Care and Treatment services of the HIV and AIDS for Key Populations in the Health Sector* to ensure that key populations had access to high-quality, evidence-based services. The strategy was gradually implemented in 22 health facilities with plans to expand to all antiretroviral treatment (ART) sites in 2020. These guidelines provide an orientation to the HIV-related program managers and health providers on the integration of health services to serve key populations, including PWID; however, they do not include additional guidance for the focus on youth PWID.

The Mental Health Program is currently drafting a National Harm Reduction Plan based on global guidance which advocates for appropriate medication-assisted therapy; injection equipment programs and antiretroviral therapy to prevent the transmission of HIV, viral hepatitis, and other blood-borne viruses; and also underlines the need for the full respect of the human rights and fundamental freedoms of people who use drugs [24].

### Opportunities for the integration of young PWID in the National Harm Reduction Plan: recommendations

It is imperative that the Mozambique Mental Health Program incorporate the specific needs of youth in the drafting of the National Harm Reduction Plan, given the available data about the specific vulnerability of youth and PWID.

Reaching young people before they start injecting is a window of opportunity to prevent drug initiation, HIV infection, and other sexually transmitted infections. Harm reduction strategies can benefit both HIV negative and positive young PWID to prevent HIV acquisition and ongoing transmission among those not yet infected and to maintain health, promote adherence to HIV treatment, and reduce the risk of onward transmission [25]. The school context is an excellent environment to advocate and introduce youth harm reduction approaches to prevent substance abuse [26, 27].

Evidence shows that improved access to health services for young people might help to prevent the social, educational, and economic risks of problematic substance use [4]. Moreover, special attention must also be given to the additional vulnerability of gender, such as female PWID who also report sex work in exchange for drugs [28].

The forthcoming Harm Reduction Plan should highlight the multidimensional approach to supporting young PWID since a comprehensive package of harm reduction interventions for this sub-population cannot exist in a vacuum. Strategies must promote collaboration between mental health and adolescent health programs to ensure that youth-friendly health services support young PWID with assessing critical interventions such as access to condoms, HIV counseling and testing, and sexual and reproductive health services (including diagnosis and treatment of STIs). Needle/syringe distribution programs should also be prepared to understand the specific needs of young PWID and ensure an environment free of stigma and discrimination. High coverage of integrated prevention and treatment services for youth sustained overtime is necessary to achieve lasting gains in the prevention of HIV infection in youth PWID [29].

Another important aspect is to strengthen the collaboration between community-based and health facility services for an effective coordination of comprehensive youth health programs. A monitoring and evaluation system that captures indicators that can be disaggregated for youth will help program managers and decision-makers to assess the effectiveness of interventions for this specific age-group. This strategic information should detect bottlenecks or gaps to service implementation, guide a targeted response, and provide the information necessary to evaluate the implementation and impact of the Harm Reduction Plan among youth.

Finally, the effective uptake of services by young PWID requires their meaningful engagement and leadership. Youth are more likely to adopt services when they participate in the development and implementation of policies, programs, and interventions that focus on their health and affect their lives. Youth can make substantial contributions to a stronger community-led response to the HIV epidemic when they are empowered with adequate skills and resources to contribute and participate [9].

## Conclusions

Young people face tremendous challenges to access HIV and sexual and reproductive health services, including inequalities, discrimination, exclusion, and violence. More efforts are needed to ensure that young people are not left behind. The current drafting of the National Harm Reduction Plan is an opportune time for Mozambique to consider comprehensive and integrated harm reduction programs and policies that focus on youth PWID to reduce the high-risk sexual and drug use behaviors that contribute to the growing HIV epidemic in the country. Effective interventions in this young generation may protect the public health investments for adults in the future.
